# Victimization as a Result of Non-Consensual Dissemination of Sexting and Psychopathology Correlates: An Exploratory Analysis

**DOI:** 10.3390/ijerph18126564

**Published:** 2021-06-18

**Authors:** Aina M. Gassó, Katrin Mueller-Johnson, Esperanza L. Gómez-Durán

**Affiliations:** 1 Faculty of Law, Universitat Internacional de Catalunya, 08017 Barcelona, Spain; 2 Institute of Criminology, Oxford University, Oxford OX13UL, UK; katrin.mueller-johnson@crim.ox.ac.uk; 3 School of Medicine, Universitat Internacional de Catalunya, 08017 Barcelona, Spain; elgomez@uic.es

**Keywords:** sexting, psychopathology, victimization, non-consensual dissemination, sext distribution

## Abstract

Sexting is generally known as creating, sending and/or forwarding of sexual content using electronic devices. When such content is non-consensually disseminated, it becomes a criminally relevant behavior. To date, very few empirical studies have examined the prevalence of non-consensual dissemination of sexting, and none of them have analyzed the relationship with psychopathology and further victimization outcomes. Therefore, the aims of this study were (1) to examine the prevalence of non-consensual dissemination of sexual content, (2) to analyze the prevalence of further victimization as a result of non-consensual dissemination of sexting and (3) to investigate the association between secondary victimization as a result of non-consensual dissemination of sexting and psychopathology. The sample comprised 1370 Spanish college students (73.6% female; mean age = 21.4 years; SD = 4.85) who answered an online survey about their engagement in sexting behaviors, online sexual victimization and psychopathology, measured by a sexting scale and the Listado de Síntomas Breve (LSB-50), respectively. Overall, 43 participants (3.14) were victims of non-consensual dissemination of sexting, and results showed those participants who had suffered further victimization reported higher psychopathology scores than those who were not victimized and that being victimized by an ex-partner was associated with poorer mental health outcomes in the victim. Further implications are discussed.

## 1. Introduction

Sexting can be defined as the act of creating, sending and/or forwarding nude or sexually explicit images or videos through electronic devices [1,2,3]. This social phenomenon has been getting increased media and scientific attention in the past few years, as it has been linked to risky sexual behaviors, negative consequences, poorer mental health and other forms of cybervictimization for those who engage in the behavior [4,5,6,7,8]. Scientific literature keeps on growing, nevertheless, psychopathology correlates of victimization as a result of non-consensual dissemination have not been sufficiently explored. Therefore, here, we examine non-consensual dissemination prevalence, possible secondary victimization experiences and potential correlates of the victim’s psychopathology.

Following Wolak and Finkelhor’s [1] theoretical framework, sexting behaviors can be divided into two broad categories: experimental sexting and aggravated sexting [1]. Experimental sexting includes voluntary behaviors that do not involve any criminal or victimizing actions (for instance, when a person sends their partner a naked picture voluntarily). On the other hand, aggravated sexting behaviors encompass all types of sexting that may involve criminal or abusive elements beyond the creation, sending or possession of self-produced sexual content [1]. Regardless of the self-generated sexual content’s origin (voluntary—experimental sexting, or coerced—aggravated sexting), sending self-generated sexual pictures and/or videos can become a risk for the sender [5]. Although voluntary and consensual sexting does not always materialize as a risk for being sexually victimized, once the sender shares the sexual photos or videos, the images can be used by the receiver in many different ways, some of which can be victimizing for the original sender. In that sense, the receiver can then distribute the sexual content without the person’s consent, threaten the person to distribute them in exchange for money or economic retribution or threaten them in exchange for more sexual content. Engaging in sexting behaviors can become a threshold for other forms of online sexual victimization such as sexting coercion, the non-consensual dissemination of sexual content, revenge porn or sextortion [5,9].

The non-consensual dissemination of sexual content refers to the distribution of a person’s sexually explicit photos or videos (taking into account both voluntarily self-produced content and non-voluntarily produced content) without the person’s consent [10]. This behavior is criminalized in most countries, and in Spain, it is criminalized under article 197.7 of the Spanish Penal Code since 2015. Article 197.7 of the Spanish Penal Code criminalizes those who, having created or directly received intimate or sexual content of a third person with their consent, disseminate, forward or give away those images or videos without the person’s consent and sets the punishment as up to one year in prison. For instance, the media brought their attention to a Spanish case where someone disseminated a sexual video of a 32-year-old woman, who ended up committing suicide as a result of the dissemination (https://elpais.com/sociedad/2019/05/29/actualidad/1559112195_230127.html) (accessed on 20 May 2021). The case has been closed by the court since they have not been able to establish authorship of the dissemination; however, as previously stated, this behavior is considered a criminal offence under the Spanish Penal Code [11]. On the other hand, in February 2020 the Spanish Supreme Court sentenced a man who had shared a friend’s naked picture without her permission [12].

The non-consensual dissemination of sexting has recently started to receive different names such as revenge porn or image-based sexual abuse [13,14]. Revenge porn is a term used to address the non-consensual dissemination of sexual content as a form of revenge in an intimate-partner or ex-partner relationship; however, it is restricted to dissemination with intentions of revenge and fails to include many other scenarios where non-consensual dissemination can take place. Henry et al. [14] state that the term revenge porn is not broad enough to explain the non-consensual dissemination of sexting, taking into account that not all of the non-consensual dissemination happens in the context of revenge, and they recommend that this term only be used when the dissemination happens inside an intimate-partner dynamic and with the intention of getting revenge. Furthermore, these authors state that the term is not appropriate since it does not take into account those cases where images have been taken without the person’s consent and the negative impact of this form of abuse on the victims [14]. For these reasons, recent research has started to use a new term known as image-based sexual abuse, which refers to the production, dissemination and/or threat to disseminate sexual images of a person without their consent [15].

Regarding the prevalence rates of non-consensual dissemination of sexting or image-based sexual abuse, results up to date are mixed, taking into account different sample sizes, definitions and instruments used to measure the phenomenon [16]. The reported prevalence for non-consensual dissemination of sexting ranges from 1% to 23% [2,17,18,19,20,21,22]. The first research to examine the non-consensual sharing of sexually explicit messages in the UK surveyed 391 young adults and found that 16.37% of the sample had perpetrated non-consensual sharing of pictures, and 21.51% of its participants had experienced victimization of non-consensual sharing of messages [23]. Henry et al. [14] surveyed 4274 Australian adults and reported that 1 out of 10 participants had sent sexual images to someone, and the content had then been distributed without their consent, and Powell et al.’s [24] results showed that 11% of their 4053 people strong Australian sample had reported some form of image-based sexual abuse perpetration at least once in their life. However, it is possible that non-consensual dissemination of sexting or image-based sexual abuse prevalence rates might be even higher, given that victims may not be aware that their sexual images have been distributed [25].

Similarly to other forms of sexual victimization, the non-consensual dissemination of sexting is expected to have a negative impact on victims’ mental health [11], and it has been linked by the media to several suicide cases (https://www.chicagotribune.com/columns/heidi-stevens/ct-heidi-stevens-tuesday-channing-smith-suicide-intimate-texts-1001-20191001-ngtjty6jpfdhxcyiv662ucts2q-story.html) (https://www.bbc.co.uk/bbcthree/article/b80a18ed-e1e9-4fc5-9400-afba5f736c9d) (accessed on 31 May 2021) [4]. Furthermore, literature has examined the association between psychopathology and other forms of victimization such as sexting coercion, revenge porn or cyberbullying, finding that participants who suffer online victimization report poorer mental health than non-victimized participants [8,26,27,28,29]. For instance, it has been reported that cyberbullying in adolescents has the same or even greater negative effects on mental health than traditional bullying, and there is a higher correlation with suicidal behavior patterns and depression than with traditional bullying [30,31]. Other than the media reports about non-consensual dissemination of sexting, to date and to our knowledge, there has not been any scientific research in Spain looking at the association between the non-consensual dissemination of sexting and psychopathology.

For the purpose of this study, we conducted a survey assessing non-consensual dissemination of sexting and subsequent forms of victimization that people could have suffered as a result and examining the presence of psychopathology using a clinically validated scale.

We hypothesize that online sexual victimization behaviors such as the non-consensual dissemination of sexting show a high prevalence among Spanish university students, as previous literature has shown [2]. Furthermore, it has been previously reported that those who sext or have had their sexting content disseminated suffer from other forms of victimization such as bullying or cyberbullying [32], so we hypothesize that non-consensual dissemination of sexting can be followed by subsequent forms of secondary victimization, such as humiliation, insults, harassment or even physical aggression, which so far remain unexplored. Moreover, we hypothesize that psychopathological impact increases as victimization experiences increase [8].

Therefore, the aims of this study were (a) to examine the prevalence of non-consensual dissemination of sexting among a Spanish college sample, (b) to explore whether victims of non-consensual dissemination of sexting experience secondary victimization as a result of non-consensual dissemination of sexting and (c) to analyze the relationship between those victimization outcomes and victim psychopathology. Since online sexual victimization has been previously associated with poorer mental health [8], we expect that participants experiencing non-consensual dissemination of sexting will score higher on the psychopathology instruments than those not having experienced non-consensual dissemination of sexting (i.e., have poorer mental health), and further, that those who suffer secondary victimization as a result of the non-consensual dissemination of sexting will report even poorer mental health than those victims of non-consensual dissemination of sexting who did not suffer any secondary further victimization.

## 2. Materials and Methods

### 2.1. Participants

The focal subsample in this study were people who participated in a large online survey on sexting behaviors and reported that they had experienced non-consensual dissemination of sexting. Out of the total sample recruited for the research (*n* = 1370), only 43 participants were victims of non-consensual dissemination of sexting. Thus, only those 43 participants answered questions regarding victimization as a result of the non-consensual dissemination of sexting. The small sample size of the subsample is a limitation, so extrapolation of results should be cautiously done, and, in order to reach more representative conclusions, a larger sample size should be used in future research.

The total sample comprised 1370 Spanish college students (both undergraduate and postgraduate students, such as Master students), including 999 women (73.6%) and 359 men (26.2%). This study was developed using undergraduates and college students because previous research has shown that adults aged between 18 and 35 years old are more likely to engage in sexting and be victimized than adults from general community samples [2]. Ages ranged from 18 to 64 years old, with a mean age of 21.40 years (SD = 4.90). The subsample (of participants who were victims of non-consensual dissemination of sexting) comprised 43 Spanish college students including 32 women (74.4%) and 11 men (25.6%). Ages ranged from 18 to 40 years old, with a mean age of 20.7 years (SD = 3.7). The descriptive statistics for the demographic variables for the total sample and subsample can be found in Table 1.

### 2.2. Procedure

The survey was administered online. After approval by the Ethics Committee of the International University of Catalunya (UIC Barcelona), with ethical approval code DRET-2018–02, the survey link was sent to university professors from Spanish universities with a request to pass it on to their students. The participating students then self-selected to take part in their own time, and no compensation was offered for participating. Before starting the online survey, participants were informed about the nature of the study and the objectives, the institution and researcher leading the study, they were given information on the measured behaviors, assured of anonymity and confidentiality and provided with contact numbers and community resources in case of concern or distress. Furthermore, participants’ consent was collected explicitly, with participants having to actively tick a box as a sign of acceptance to start the survey after reading the information about the study.

The questionnaire took approximately 20–25 min to complete, and once completed, students were again given information on community resources in case of distress and the email address to contact the investigators in case of concerns. No participant contacted the investigators.

### 2.3. Instruments

#### 2.3.1. Sexting Questionnaire

For the purpose of this study, sexting was defined as creating, sending and/or forwarding nude or sexually explicit images or videos through any electronic device [3]. We created a sexting scale based on the JOV-Q [33] to assess four different types of sexting behaviors. For each of the measured sexting behaviors, we asked how many times participants had engaged in the behavior in the past year. This then was recoded as yearly prevalence (Yes, at least once in the past year/No, never engaged in this behavior in the past year). The four sexting-related items we measured were (1) creating and sending your own nude pictures or sexual content, (2) being a victim of non-consensual dissemination of sexual content, (3) being pressured to sext and (4) being threatened to sext.

For the purpose of the present study, those participants that had responded “yes” to item 2 (being a victim of non-consensual dissemination of sexual content) were requested to answer a secondary victimization question, where five different victimization outcomes that victims could have experienced as a result of the non-consensual dissemination of sexting were assessed. The question these participants had to answer was as follows: As a result of being a victim of non-consensual dissemination of your sexual content, have you suffered any of the following actions: (1) someone made fun of you; (2) someone insulted you; (3) someone humiliated you; (4) someone physically harmed you and/ or (5) someone harassed you. For each of these questions, participants were asked to specify through what modus the victimization was received (online/offline) and who was the perpetrator (stranger, friend, partner or/and ex-partner). We also differentiated the victimization outcomes by perpetrator: victimization by stranger, victimization by friend, victimization by partner and victimization by ex-partner. Finally, we grouped the different victimization outcomes by severity in order to create two different secondary victimization categories: less severe secondary victimization, which included being made fun of and being insulted, and severe secondary victimization, which included humiliation, physical aggression and harassment. For the purpose of this research, “being made fun of” and “being insulted” were considered as less severe forms of victimization because they have a less aggressive connotation and can be carried out more privately than the other forms of victimization. “Humiliation”, “physical aggression” and “harassment” have a more degrading and aggressive connotation and, thus, were considered as more severe forms of victimization.

#### 2.3.2. Mental Health Questionnaire

In order to measure mental health, we used the Spanish version of LSB-50, which is a revised and shorter version of the SCL-90-R, one of the most reliable and clinically validated psychopathology scales available in Spain [34]. We used the short version to avoid participant fatigue. This instrument consists of 50 items that assess psychopathological symptomatology. Responses to the items were collected on a five-point Likert scale (0 = never and 4 = extremely). We used the global psychopathology subscale, the depression subscale and the anxiety subscale for this study. To analyze the presence or absence of mental health symptoms, the results obtained from the LSB-50 questionnaire were converted according to the authors’ guidelines [34].

#### 2.3.3. Sociodemographic Questionnaire

We included questions about age, sex, marital status, parental marital status, place of residence, employment situation, academic situation and questions about frequency and use of phones and social media.

### 2.4. Data Analysis

All analyses were conducted using IBM SPSS V.25. The performed analyses for different variables were as follows: frequency and percentage for qualitative variables, mean and standard deviation when normal quantitative variables were measured and median and quartiles for quantitative non-normal variables. To compare non-normal variables, we performed separate analysis using Mann–Whitney test for two groups and Kruskal–Wallis test for more than two groups. To check the normality, the tests of Kolmogorov–Smirnov and Shapiro–Wilk were performed. Due to group size limitations, when analyzing secondary victimization, secondary victimization outcomes were dichotomized as secondarily victimized vs. not secondarily victimized or severe secondary victimization vs. non-severe victimization. Separate analyses were conducted for each of the psychopathology variables (IGS, depression and anxiety) and the measured independent variables. All the tests were two-tailed and were significant under 0.05.

## 3. Results

### 3.1. Prevalence of Secondary Victimization as a Result of the Non-Consensual Dissemination of Sexting

Out of the 1370 original sample, 43 participants (3.14%) were victims of non-consensual dissemination of sexting, and out of the subsample of people who had been victimized, 20.9% (9 participants) had experienced secondary victimization online as a result of the non-consensual dissemination of sexting, whilst 11.6% (5 participants) had suffered offline victimization as a result of the non-consensual dissemination of sexting. Results regarding who the victimization outcome was carried out by (perpetrator) showed that overall, most victims of non-consensual dissemination of sexting suffered from victimization outcomes carried out by a stranger, rather than by a friend, boyfriend or ex-partner. When reading and interpreting these results, the small size of the subsample should be taken into account. Prevalence rates of being victimized as a result of the non-consensual dissemination of sexting by the modus and perpetrator are shown in Table 2.

### 3.2. Victimization and Psychopathology Scores

**Figure 1:**
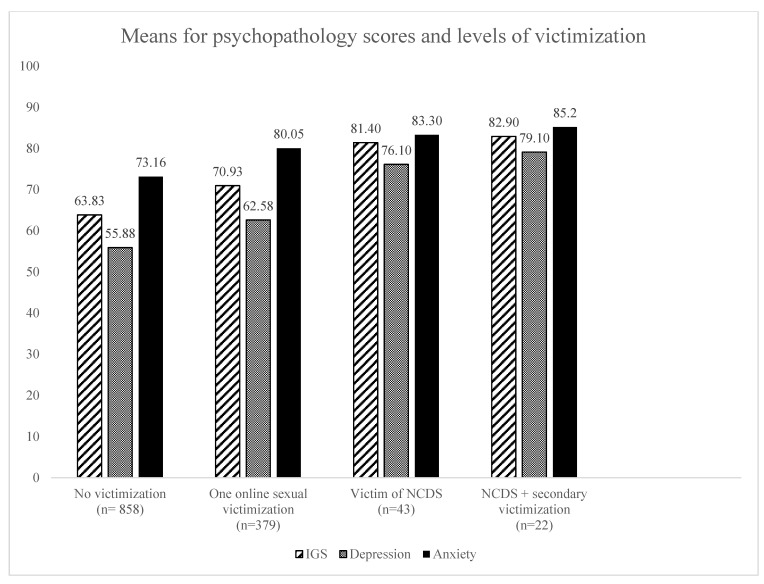
Means for psychopathology scores and levels of victimization. Note: NCDS = non-consensual dissemination of sexting.

Out of the total sample (*N* = 1370), 62.6% of participants did not suffer online sexual victimization, 27.7% suffered from at least one form of online sexual victimization, 3.14% suffered from non-consensual dissemination of sexting and 1.6% suffered from secondary victimization as a result of the non-consensual dissemination of sexting. When exploring the association between these different levels of victimization and psychopathology scores, Kruskal–Wallis test showed significant results for the three psychopathology measures and the four levels of victimization (no victimization, being victim of one form of victimization, being victim of non-consensual dissemination of sexting and suffering from secondary victimization as a result of non-consensual dissemination of sexting): for global psychopathology, H(3) = 36.05, *p* < 0.000; for depression, H(3) = 28.753, *p* < 0.000; and for anxiety, H(3) = 23.78, *p* < 0.000, thus showing that psychopathology scores increase, as the number of victimizations suffered increases. Means scores for these results are shown in Figure 1; however, these results should be interpreted as exploratory, taking into account the size of the subsample (*N* = 43).

### 3.3. Secondary Victimization by Perpetrator and Psychopathology Scores

Out of the total subsample (*N* = 43), 51% (*N* = 22) suffered from non-consensual dissemination of sexting and secondary victimization as a result. Although there was a tendency for psychopathology scores to be higher for those who suffered secondary victimization as a result of non-consensual dissemination of sexting than for those who suffered non-consensual dissemination of sexting without further related victimization, these differences were not statistically significant (global psychopathology: U = 174.0, *p* = 0.805; depression: U = 177.5, *p* = 0.717; anxiety: U = 182.5, *p* = 0.612). For victims of non-consensual dissemination of sexting who suffered secondary victimization, mean scores were as follows: global psychopathology M = 82.9 (SD = 22.26), depression M = 79.1 (SD = 22.74), anxiety M = 85.2 (SD = 20.9); whilst for victims of non-consensual dissemination of sexting without further victimization outcomes mean scores were global psychopathology M = 80.8 (SD = 24.39), depression M = 75.0 (SD = 27.36), anxiety M = 82.7 (SD = 20.15). These results should be cautiously extrapolated, and further research with bigger samples should further confirm these data.

Regarding psychopathology scores and perpetrator figures, our results showed that psychopathology scores were higher for participants who had been secondarily victimized by ex-partners, than for any of the secondary victimization forms perpetrated by other people. Moreover, scores were higher for global psychopathology than for anxiety or depression. Means for psychopathology scores for secondary victimization perpetrated by an ex-partner were as follows: global psychopathology M = 94.5 (SD = 5.20), depression M = 76.0 (SD = 22.52), anxiety M = 95.0 (SD = 3.74); for secondary victimization perpetrated by a friend they were global psychopathology M = 80.1 (SD = 28.84), depression M = 72.0 (SD = 25.31), anxiety M = 82.1 (SD = 24.48); and for secondary victimization perpetrated by a stranger they were global psychopathology M = 82.2 (SD = 24.59), depression M = 73.5 (SD = 27.32), anxiety M = 85.2 (SD = 19.57). Furthermore, results from non-parametric Mann–Whitney U test showed a significant relationship between suffering secondary victimization from a stranger or from an ex-partner and psychopathology scores; however, the size of the subsample should be kept in mind when interpreting these results. Results are depicted in Table 3.

### 3.4. Secondary Victimization by Modus and by Severity and Psychopathology

Results indicate higher psychopathology scores for suffering secondary victimization both online and offline versus those participants who had suffered secondary victimization either online or offline, although the small subsample size did not allow us to explore this further. For suffering secondary victimization online mean psychopathology scores were as follows: global psychopathology M = 86.4 (SD = 16.92), depression M = 81.9 (SD = 18.71), anxiety M = 88.9 (SD = 16.06); for suffering secondary victimization offline mean psychopathology scores were global psychopathology M = 84.4 (SD = 27.89), depression M = 82.2 (SD = 27.60), anxiety M = 86.2 (SD = 25.86); and for suffering secondary victimization both online and offline mean psychopathology scores were global psychopathology M = 96.0 (SD = 5.20), depression M = 92.7 (SD = 10.97), anxiety M = 98.0 (SD = 1.73).

Finally, results from the Mann–Whitney U test, when comparing psychopathology scores for those who had suffered severe secondary victimization, in comparison to those without secondary victimization, showed a significant association between severe victimization outcomes (humiliation, harassment and physical aggression) and all psychopathology measures (global psychopathology U(2) = 15.672, *p* = 0.0012; depression U(2) = 14.0.943, *p* = 0.00412; anxiety U(2) = 14.2944, *p* = 0.01402). Results are presented in Table 4.

## 4. Discussion

In this study, we assessed whether Spanish college students had been victims of non-consensual dissemination of their sexting content and whether they had suffered from secondary victimization outcomes as a result of the non-consensual dissemination of sexting. Finally, we wanted to explore whether any relationship exists between those variables and psychopathology. In order to do so, we conducted an online survey using a sexting scale adapted from the JOV-Q and validated measures of mental health (LSB-50). To our knowledge, this is the first empirical research investigating the mental health correlates of secondary victimization as a result of the non-consensual dissemination of sexual content.

Overall, our findings suggest that 3.14% out of the total sample (*N* = 1370) had been victims of non-consensual dissemination of sexting. Out of those participants who had been victimized, some suffered secondary victimization because of the dissemination of their sexual content. Our results suggest that, between 11% and 21% of the subsample had suffered from some type of victimization as a result of the non-consensual dissemination of sexting and that those victimization outcomes occurred either online, offline or both and that they could be perpetrated by different people (stranger, friend, partner or ex-partner). Our results indicate that, in general, victims who had had their content disseminated reported more online secondary victimization as a result of the non-consensual dissemination of sexting than offline or both. These results support previous literature [30,35,36]. Taking into account the technological and online nature of sexting and non-consensual dissemination of sexting, results showing higher reported rates of secondary online victimization were expected. However, and regardless of the small size of the subsample (*N* = 43), reported prevalence rates indicate that non-consensual dissemination of sexting can also result in secondary offline victimization outcomes, highlighting the need for well-defined prevention campaigns [37].

Our results show a significant association between being a victim of non-consensual dissemination of sexting and all psychopathology measures, but our sample also experienced other forms of online sexual victimization (being pressured or threatened to sext). Up to 27.7% suffered from at least one form of online sexual victimization, and the 3.14% suffered from two or more forms of online sexual victimization. According to our results, the more types of victimization a person suffers, the higher the reported psychopathology scores. These results corroborate a linear relationship between polyvictimization and poorer mental health as previously suggested by other authors [38,39].

Furthermore, our results show that experiencing secondary victimization as a result of the non-consensual dissemination of sexting is associated with higher psychopathology scores, highlighting the need to protect those who suffer non-consensual dissemination of sexting even after the initial victimization has occurred. Secondary victimization as a result of the non-consensual dissemination of sexting by a stranger was more prevalent than being victimized by any other person, in line with previous research [40]. In the absence of other empirical literature on this matter, these findings may make sense when we contextualize the phenomenon of sexting and non-consensual dissemination of sexting. Wolak et al. [41] reported that 90% of the online victimizations were carried out by strangers, and Reyns et al. [40] found higher online victimization behaviors perpetrated by strangers than by intimate partners or friends.

When examining the link between the victim–perpetrator relationship and psychopathology, we found that people victimized by a stranger and by an ex-partner reported significantly higher percentages of meeting the clinical threshold on the global psychopathology subscale than those victimized in other constellations. However, and even though reported prevalence rates were higher for being victimized by a stranger, when analyzing the association between secondary victimization and psychopathology, results showed that participants who had been victimized by an ex-partner showed higher psychopathology scores than those who had been victimized by a friend or a stranger. These results support previous results, given that victimization by someone you are emotionally attached to might have a bigger impact on emotional stability than being victimized by a stranger [42].

Finally, the intensity of the victimizing behavior also proved to be relevant. Although secondary online victimization was most prevalent in our secondarily victimized sample, offline victimization was also described and showed similar psychopathological scores to online victimization outcomes. Victims of both online and offline secondary victimization appeared to report even higher psychopathology scores (although subsample size was too small to allow significance testing). Furthermore, the severity of the secondary victimization derived from the non-consensual dissemination of sexting was categorized into two groups: less severe victimization (insulting and making fun) and severe victimization (humiliation, harassment and physical aggression) as previous literature has done [39]. Our results showed significant differences in global psychopathology and depression between those participants who suffered severe secondary victimization as a result of the non-consensual dissemination of sexting and those who suffered no or less severe secondary victimization. These findings are in line with previous research, since it would be expected that the more severe the victimization is, the higher the reported psychopathology scores [39].

## 5. Conclusions

In summary, our findings indicate that out of all of the people from our sample, only 3.14% of participants had had their nude images or sexual content disseminated without their consent in the past year, showing lower prevalence rates than reported rates for other forms of online sexual victimization [38]. The main contributions of the present study are reporting that victims of non-consensual dissemination of sexting suffer a psychopathological impact are often secondarily victimized as a result of the non-consensual dissemination suffered, which increases their suffering, and that they are more likely to be secondarily victimized online than offline, with both modi of victimization being equally harmful according to psychopathological scores but increasing their harmful potential when both occur. The psychopathological impact was also higher in the most severe forms of victimization outcomes. Furthermore, this study found that participants reported suffering more secondary victimization as a result of the non-consensual dissemination of sexting by a stranger than by a friend or ex-partner, even though being victimized by an ex-partner was associated with higher psychopathology scores than being victimized by any other person.

We believe that, despite the exploratory nature of the study and the small sample size of victims of non-consensual dissemination, our results provide new and relevant knowledge that can help develop new and better targeted prevention campaigns. Furthermore, due to the expected negative impact on victims’ well-being and the potential contribution that could be made to prevent offline crime involved in secondary victimization, our results support the need to update penal codes worldwide, if not done yet, by including the criminalization of non-consensual dissemination of sexual content.

This study has several limitations that should be taken into account. First, this study is cross-sectional, so no causal relationships can be established between the mental health variables and the studied behaviors and outcomes. Furthermore, it should be noted that participants reported yearly prevalence of non-consensual dissemination of sexting, however, mental health symptoms were reported for the previous weeks, so, again, no causal relationship can be established from our results. Secondly, the sample used was composed of only college students, rather than the general population, so extrapolation of results should be cautiously done. The sample was self-selected via an online survey, thus the ratio of males to females is not even, and other bias in the sample selection might affect the results. It would be important that other studies replicate these results with other mental health measures and with bigger sample sizes. Finally, the small sample size of the subsample is a limitation, so extrapolation of results should be cautiously done, and, in order to reach more representative conclusions, a larger sample size should be used in future research. Lastly, given the scarceness of the published data so far, our study contributes toward bridging the gap in existing literature. It highlights the importance of looking beyond the prevalence of the initial victimization to further understand the actual consequences these victims face and to develop effective interventions and support for this group.

## Figures and Tables

**Table 1:** Descriptive statistics of demographic and background variables for the total sample and subsample.

Demographic Variables	Total Sample % (*n* = 1370)	Victims of Non-Consensual Dissemination of Sexting % (*n* = 43)
Gender		
Male	26.2	25.6
Female	73.6	74.4
Age	21.4 (4.9)	20.7 (3.7)
Marital Status		
Single	54.6	51.2
In relationship	42.0	46.5
Married	1.20	2.3
Parental Marital Status		
Married	71.3	61.9
Divorced/separated	22.5	26.2
Widow	4.40	7.1
Other	1.80	4.8
Academic Situation		
Undergraduate	92.4	88.4
Master’s degree	4.00	4.7
Erasmus/international student	1.5	2.3
Other	2.20	4.7
Living Situation		
With parents	62.4	54.8
Student apartment	22.4	35.7
Off-campus student residence	4.60	4.8
On-campus student residence	0.70	0
Alone	3.80	0
With partner	6.20	4.8
Employment Status		
Unemployed	67.4	60.5
Employed full time	5.10	0
Employed partial time	27.4	39.5
Own Smartphone	98	97.7
Age at First Phone Ownership	13.9 (3.4)	13.1 (2.1)
Age at First Internet Access	12.01 (3.8)	11.1 (2.4)
Internet Access		
Mobile phone	89.8	90.7
Laptop	27.8	34.9
Desktop PC	6.0	2.3
Tablet	30.9	27.9
PlayStation	5.7	2.3
Frequency of Internet Access		
Once a week	0.1	0
2–3 times a week	0.40	0
Everyday	33.0	38.1
2–3 h per day	16.7	14.3
More than 3 h per day	48.0	47.6
Social Media Use		
Yes	97.8	100

**Table 2:** Prevalence rates for the forms of secondary victimization as a result of non-consensual dissemination of sexting by modus and perpetrator.

Forms of Secondary Victimization	Modus of Victimization				Perpetrator *			
	*n*	Any Online % (*n*)	Any Offline % (*n*)	Both % (*n*)	*n*	Stranger % (*n*)	Friend % (*n*)	Ex-Partner % (*n*)
Being made fun of	40	27.9 (11)	23.3 (9)	11.6 (5)	33	54.5 (18)	57.6 (19)	15.2 (5)
Being insulted	33	27.9 (9)	11.6 (4)	9.3 (3)	28	60.7 (17)	46.4 (13)	25 (7)
Humiliation	34	25.6 (9)	23.3 (8)	14 (5)	31	54.8 (17)	25.8 (8)	22.6 (7)
Physical aggression	10	-	4.7 (1)	-	19	15.8 (3)	15.8 (3)	10.5 (2)
Harassment	29	20.9 (6)	11.6 (3)	7 (2)	27	51.9 (14)	22.2 (6)	22.2 (6)

* Perpetrator = participants who responded affirmatively to the victimization questions were asked who the victimization was perpetrated by.

**Table 3:** Median and quartiles in parentheses for psychopathology scores and perpetrator of secondary victimization.

	*N*	IGS Scores Median (Q1–Q3)	Depression Scores Median (Q1–Q3)	Anxiety Scores Median (Q1–Q3)
Secondary victimization by stranger				
No	1321	75 (45–90)	60 (35–85)	80 (55–95)
Yes	13	90 (75–97.5)	85 (50–94)	95 (75–98.5)
Significance test (Mann–Whitney U)		*U* = 11.72, *p* = 0.023	*U* = 11.21, *p* = 0.058	*U* = 11.27, *p* = 0.053
Secondary victimization by friend				
No	1323	75 (45–90)	60 (35–85)	80 (55–95)
Yes	11	90 (70–99)	80 (45–99)	95 (75–99)
Significance test (Mann–Whitney U)		*U* = 9.93, *p* = 0.037	*U* = 9.30, *p* = 0.111	*U* = 9.25, *p* = 0.120
Secondary victimization by ex-partner				
No	1330	75 (45–90)	60 (35–85)	80 (55–95)
Yes	4	94.5 (90–99)	80 (53.8–94.3)	95.5 (91.3–98.3)
Significance test (Mann–Whitney U)		*U* = 4.48, *p* = 0.018	*U* = 3.55, *p* = 0.247	*U* = 4.25, *p* = 0.039

**Table 4:** Median and quartiles in parentheses for psychopathology scores and severity of secondary victimization.

Severe Secondary Victimization		IGS Scores	Depression Scores	Anxiety Scores
Yes	16	95.5 (90–99)	87.5 (52.5–99)	95.5 (78.8–99)
No	1334	75 (45–90)	60 (35–85)	80 (55–95)
Significance test (Mann–Whitney U)		*U* = 15.67, *p* = 0.001	*U* = 14.94, *p* = 0.004	*U* = 14.29, *p* = 0.014

## Data Availability

The data used to support the findings of this study are available from the corresponding author upon request.
